# Estimated plasma volume status: association with congestion, cardiorenal syndrome and prognosis in precapillary pulmonary hypertension

**DOI:** 10.3389/fcvm.2023.1161041

**Published:** 2023-05-10

**Authors:** Athiththan Yogeswaran, Manuel J. Richter, Faeq Husain-Syed, Zvonimir Rako, Natascha Sommer, Friedrich Grimminger, Werner Seeger, Hossein Ardeschir Ghofrani, Henning Gall, Khodr Tello

**Affiliations:** ^1^ Department of Internal Medicine, Universities of Giessen and Marburg Lung Center (UGMLC), Institute for Lung Health (ILH), Cardio-Pulmonary Institute (CPI), Member of the German Center for Lung Research (DZL), Giessen, Germany; ^2^ Department of Internal Medicine, Justus-Liebig-University Giessen, Giessen, Germany

**Keywords:** pulmonary arterial hypertension, chronic thromboembolic pulmonary hypertension, glomerular filtration rate, fluid balance, survival

## Abstract

**Background:**

Volume overload is often associated with clinical deterioration in precapillary pulmonary hypertension (PH). However, thorough assessment of volume overload is complex and therefore not routinely performed. We examined whether estimated plasma volume status (ePVS) is associated with central venous congestion and prognosis in patients with idiopathic pulmonary arterial hypertension (IPAH) or chronic thromboembolic PH (CTEPH).

**Methods:**

We included all patients with incident IPAH or CTEPH enrolled in the Giessen PH Registry between January 2010 and January 2021. Plasma volume status was estimated using the Strauss formula.

**Results:**

In total, 381 patients were analyzed. Patients with high ePVS (≥4.7 vs. <4.7 ml/g) at baseline showed significantly increased central venous pressure (CVP; median [Q1, Q3]: 8 [5, 11] mmHg vs. 6 [3, 10] mmHg) and pulmonary arterial wedge pressure (10 [8, 15] mmHg vs. 8 [6, 12] mmHg), while right ventricular function was not altered. In multivariate stepwise backward Cox regression, ePVS was independently associated with transplant-free survival at baseline and during follow-up (hazard ratio [95% confidence interval]: 1.24 [0.96, 1.60] and 2.33 [1.49, 3.63], respectively). An intra-individual decrease in ePVS was associated with a decrease in CVP and predicted prognosis in univariate Cox regression. Patients with high ePVS without edema had lower transplant-free survival than those with normal ePVS without edema. In addition, high ePVS was associated with cardiorenal syndrome.

**Conclusions:**

In precapillary PH, ePVS is associated with congestion and prognosis. High ePVS without edema may represent an under-recognized subgroup with poor prognosis.

## 1. Introduction

Pulmonary hypertension (PH) is a devastating vascular disease characterized by increased pulmonary arterial pressure and resistance (1). The right ventricle adapts to the increased right ventricular afterload through various mechanisms such as hypertrophy and increased contractility (2). However, even slight changes in pulmonary hemodynamics can unbalance the fragile interaction between the right ventricle and the pulmonary artery, resulting in so-called uncoupling.

One of the most common causes of the previously described congestion and, therefore, exacerbations of PH is plasma volume overload (3). However, optimal adjustment of the patient's fluid status is time-consuming and complex, since it currently depends on parameters such as the diameter of the inferior vena cava or even the invasive measurement of central venous pressure (CVP) by right heart catheterization (4). Clinical signs such as edema or body weight gain are considered uncertain signs of volume overload (5, 6). A volume overload assessment is therefore not always performed, especially in an ambulatory setting where clinicians sometimes rely only on the brain natriuretic peptide (BNP) concentration to indicate right ventricular stress.

Recent data suggest that the plasma volume status can be estimated using different formulas (7, 8). In patients with left heart failure, the estimated plasma volume status (ePVS) is associated with prognosis, and the intra-individual change in estimated plasma volume can be used to monitor congestion (8). However, the utility and prognostic relevance of ePVS in patients with precapillary PH are not yet fully understood. Thus, we aimed to assess the clinical relevance of (i) edema and (ii) ePVS in patients with idiopathic pulmonary arterial hypertension (IPAH) or chronic thromboembolic PH (CTEPH).

## 2. Methods

### 2.1. Study population

All consecutive patients with newly diagnosed IPAH (PH classification group 1) or CTEPH (PH classification group 4) who were enrolled in the prospectively recruiting Giessen PH registry (9) between January 2010 and January 2021 were included in this study. Patients without right heart catheterization or hemoglobin/hematocrit values at the time of diagnosis were excluded from further analysis. The diagnosis was made by a multidisciplinary panel of physicians, surgeons, and radiologists. All patients gave their written informed consent to be enrolled in the Giessen PH registry (and subsequent analyses of registry data). The study was approved by the local ethics committee of the University of Giessen (#266/11).

### 2.2. Right heart catheterization

Right heart catheterization was performed as previously described (10). Briefly, a sheath was placed in the right internal jugular vein under local anesthesia. In rare cases, the left internal jugular vein or femoral vein was used. CVP, pulmonary arterial pressure, and pulmonary arterial wedge pressure (PAWP) were measured during stopped expiration using a Swan-Ganz catheter (11). Cardiac output was determined with the direct Fick method and thermodilution, and was used to calculate pulmonary vascular resistance {[mean pulmonary arterial pressure (mPAP)—PAWP]/cardiac output}. If oxygen consumption was not measurable, resting oxygen uptake was estimated using the LaFarge and Bergstra formula (12).

### 2.3. Echocardiography, laboratory measurements, and estimation of plasma volume status

Echocardiography was performed by experts according to the guidelines that were valid at the time of the measurement (1, 13, 14). The echocardiographic assessment included, among others, the systolic excursion of the tricuspid annular plane (TAPSE) and the right atrial area. The laboratory measurements included, among others, BNP and creatinine levels and were performed in the central laboratory of the University Hospital Giessen. Glomerular filtration rate was estimated (eGFR) using the Chronic Kidney Disease Epidemiology Collaboration formula (142 * min[serum creatinine/kappa, 1]^alpha^ * max[serum creatinine/kappa, 1]^−1.2^ * 0.9938^Age^ * Sex Factor, where alpha = −0.241 [female] or −0.302 [male], kappa = 0.7 [female] or 0.9 [male], and Sex Factor = 1.012 [female] or 1 [male]) (15, 16).

Plasma volume status was estimated at baseline and during follow-up using the Strauss formula: ePVS = (1-hematocrit)/hemoglobin (g/dl) * 100 (8). This formula considers hematocrit and hemoglobin concentration as estimators for plasma volume status. Both hematocrit and hemoglobin concentration are known predictors of cardiovascular events (17, 18). In addition, the relative change of ePVS can be determined by the following formula: ePVS_follow−up_–ePVS_baseline_.

### 2.4. Statistical analyses

The Shapiro-Wilk test was used to assess whether the included variables were normally distributed. Normally distributed continuous data are presented as mean ± standard deviation and *t*-tests were used to compare means between groups. Non-normal continuous data are shown as median [first quartile, third quartile] and Wilcoxon rank tests were used to compare medians between groups. Chi-square tests were used to compare categorical data between groups. We considered *p* < 0.05 to indicate statistical significance. Survival time and transplant status were censored at either 12 months (short-term transplant-free survival) or 60 months (long-term transplant-free survival). Kaplan-Meier estimators with log-rank tests and univariate and multivariate (stepwise backward) Cox regression analyses were used to examine the prognostic relevance of parameters. In the multivariate analyses, the stepwise backward model was chosen based on Akaike information criteria using the R stats package. Cox regression coefficients were used to develop new prognostic models based on independent predictors of mortality/transplant, as described previously (19). Multivariate, backward binary logistic regression was performed using a *p* value threshold of 0.10. Missing data were not imputed. Statistical analyses were performed with R version 4.0.4 (The R Foundation, Vienna) and SPSS 29.0 (IBM, Armonk, USA).

## 3. Results

### 3.1. Baseline characteristics

A total of 381 patients were enrolled in the study, of whom 145 (38%) were diagnosed with IPAH and 236 (62%) with CTEPH. The median age of the study population was 69 [55, 76] years and 217 (57%) were female. The included patients showed severe impairment of pulmonary hemodynamics (Table 1). The median CVP was 7 [4, 10] mmHg and the median ePVS was 4.14 [3.53, 4.92] ml/g.

**Table 1:** Baseline characteristics.

Parameter	All (*n* = 381)	Normal ePVS (*n* = 254)	High ePVS (*n* = 127)	*p* value
Age, years	69 [55, 76] *n* = 381	68 [54, 76] *n* = 254	70 [58, 77] *n* = 127	0.106
Female sex, *n* (%)	217 (57%) *n* = 381	124 (49%) *n* = 254	93 (73%) *n* = 127	<0.001
Diagnosis of IPAH, *n* (%)	145 (38%) *n* = 381	101 (40%) *n* = 254	44 (35%) *n* = 127	0.391
Edema, *n* (%)	147 (39%) *n* = 317	84 (33%) *n* = 215	63 (50%) *n* = 102	<0.001
Diuretic treatment, *n* (%)	221 (58%) *n* = 317	142 (56%) *n* = 215	79 (62%) *n* = 102	0.053
Hemoglobin, g/dl	14.1 [12.5, 15.5] *n* = 381	14.8 [14.1, 16.1] *n* = 254	12.1 [11.0, 12.5] *n* = 127	<0.001
Hematocrit	0.42 [0.38, 0.46] *n* = 381	0.44 [0.42, 0.49] *n* = 254	0.37 [0.34, 0.38] *n* = 127	<0.001
Weight, kg	77 [69, 89] *n* = 378	78 [69, 90] *n* = 253	76 [67, 88] *n* = 125	0.252
Height, cm	169 [163, 176] *n* = 378	170 [164, 178] *n* = 253	168 [160, 174] *n* = 125	0.005
mPAP, mmHg	41 [33, 48] *n* = 371	43 [34, 50] *n* = 248	38 [31, 46] *n* = 123	0.006
PAWP, mmHg	9 [7, 13] *n* = 369	8 [6, 12] *n* = 246	10 [8, 15] *n* = 123	<0.001
Pulmonary vascular resistance, dyn·s/cm^5^	589 [376, 830] *n* = 371	636 [419, 879] *n* = 248	454 [274, 698] *n* = 123	<0.001
CVP, mmHg	7 [4, 10] *n* = 370	6 [3, 10] *n* = 247	8 [5, 11] *n* = 123	0.027
Cardiac index, L/min/m^2^	2.32 [1.91, 2.71] *n* = 370	2.21 [1.82, 2.56] *n* = 247	2.58 [2.13, 2.92] *n* = 123	<0.001
Mixed venous oxygen saturation, %	63 [58, 69] *n* = 369	64 [59, 69] *n* = 246	62 [55, 66] *n* = 123	0.002
ePVS, ml/g	4.14 [3.53, 4.92] *n* = 381	3.80 [3.26, 4.14] *n* = 254	5.25 [4.93, 5.89] *n* = 127	<0.001
BNP, pg/ml	193 [79, 429] *n* = 342	175 [70, 389] *n* = 230	237 [113, 463] *n* = 112	0.072
eGFR, ml/min/1.73 m^2^	70 [52, 90] *n* = 377	73 [57, 92] *n* = 253	64 [43, 80] *n* = 124	<0.001
TAPSE, mm	19 ± 4 *n* = 363	19 ± 4 *n* = 244	19 ± 4 *n* = 119	0.241
TAPSE/PASP, mm/mmHg	0.294 [0.224, 0.397] *n* = 357	0.291 [0.221, 0.376] *n* = 240	0.327 [0.231, 0.444] *n* = 117	0.058
Right atrial area, cm^2^	20 [15, 25] *n* = 339	20 [16, 25] *n* = 222	19 [13, 26] *n* = 117	0.254
Inferior vena cava diameter, mm	21 ± 5 *n* = 162	21 ± 5 *n* = 107	21 ± 6 *n* = 55	0.717

Data are presented as median [Q1, Q3] or mean ± standard deviation unless otherwise specified. BNP, brain natriuretic peptide; CVP, central venous pressure; eGFR, estimated glomerular filtration rate; ePVS, estimated plasma volume status; IPAH, idiopathic pulmonary arterial hypertension; mPAP, mean pulmonary arterial pressure; PASP, pulmonary arterial systolic pressure; PAWP, pulmonary arterial wedge pressure; TAPSE, tricuspid annular plane systolic excursion.

### 3.2. Prognostic relevance of edema in incident and prevalent patients with PH

**Figure 1:**
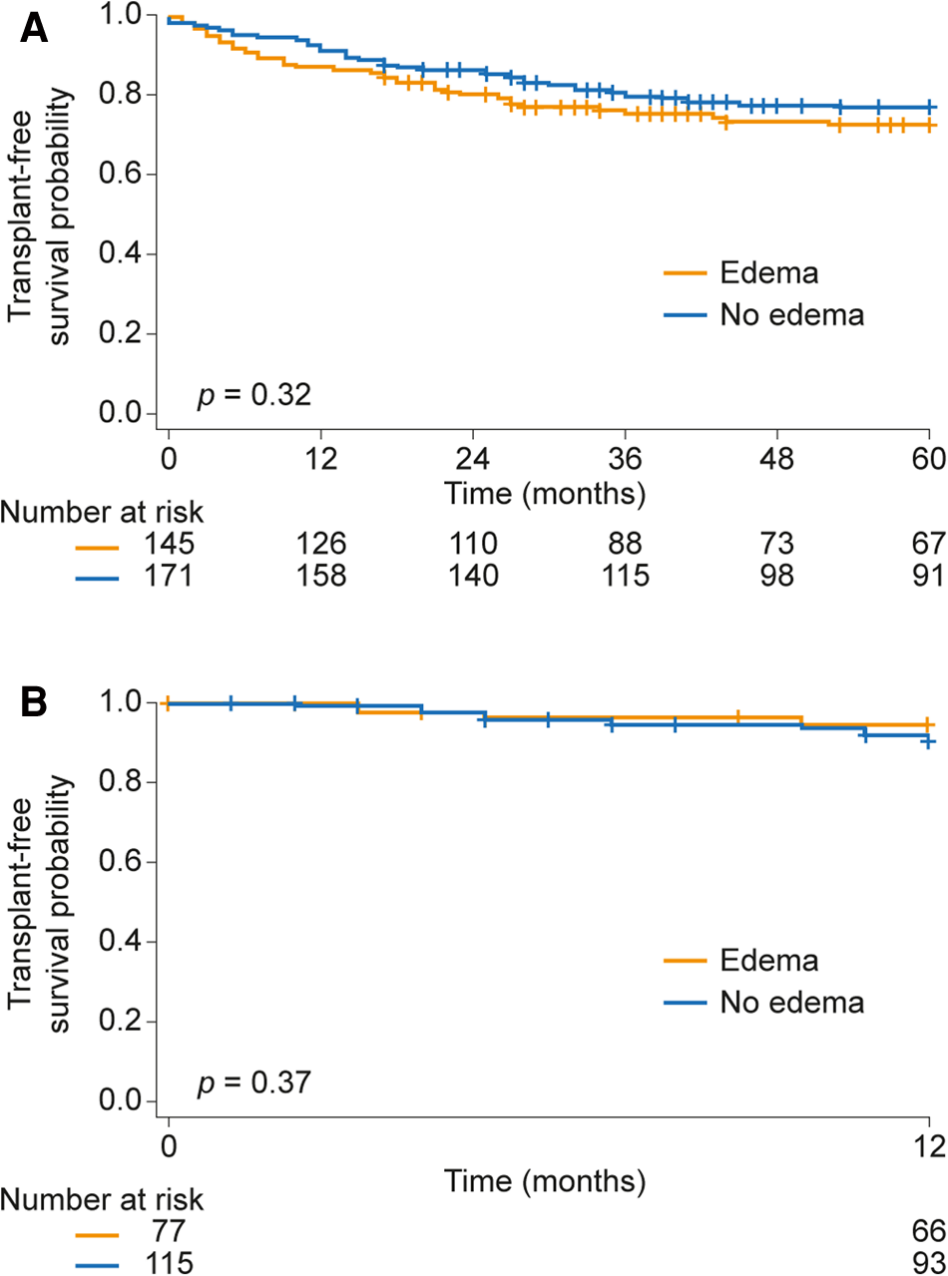
Prognostic relevance of edema (**A**) at baseline and (**B**) during follow-up in patients with precapillary pulmonary hypertension.

First, we evaluated whether the clinical assessment of edema in precapillary PH is prognostically relevant. Data on edema status were available for 317 patients at baseline (incident patients) and 154 patients at follow-up (median 8 [6, 9] months after baseline; prevalent patients). As shown in the Kaplan-Meier curves in Figure 1, patients with edema had comparable transplant-free survival rates to those without edema, both at baseline and at follow-up. In addition, there was no difference in the transplant-free survival distribution between patients whose edema resolved and those who developed edema during follow-up (Supplementary Figure S1A). In patients with edema at baseline, resolution of the edema was also not associated with increased transplant-free survival (Supplementary Figure S1B).

### 3.3. Association of high ePVS with congestion and chronic cardiorenal syndrome in incident patients with PH

Second, we investigated whether elevated ePVS is associated with congestion in patients with precapillary PH. We classified ePVS in the lowest and intermediate thirds (below 4.7 ml/g) as normal, and ePVS ≥4.7 ml/g as high.

Baseline characteristics of patients with normal and high ePVS are compared in Table 1. Interestingly, patients with high ePVS showed significantly increased PAWP and CVP, while mPAP and (concordantly) pulmonary vascular resistance were lower than in patients with normal ePVS. In turn, patients with high ePVS had edema significantly more often than those with normal ePVS. In addition, BNP levels tended to be higher and eGFR was significantly lower in patients with high ePVS compared with patients with normal ePVS. However, TAPSE, the diameter of the inferior vena cava, and right atrial area were not affected. Interestingly, patients with high ePVS had chronic cardiorenal syndrome, defined by an eGFR < 60ml/min/1.73 m^2^, significantly more often than patients with normal ePVS (44% vs. 28%, *X*^2^ *p* = 0.003).

### 3.4. Prognostic relevance of ePVS in incident patients with PH

**Figure 2:**
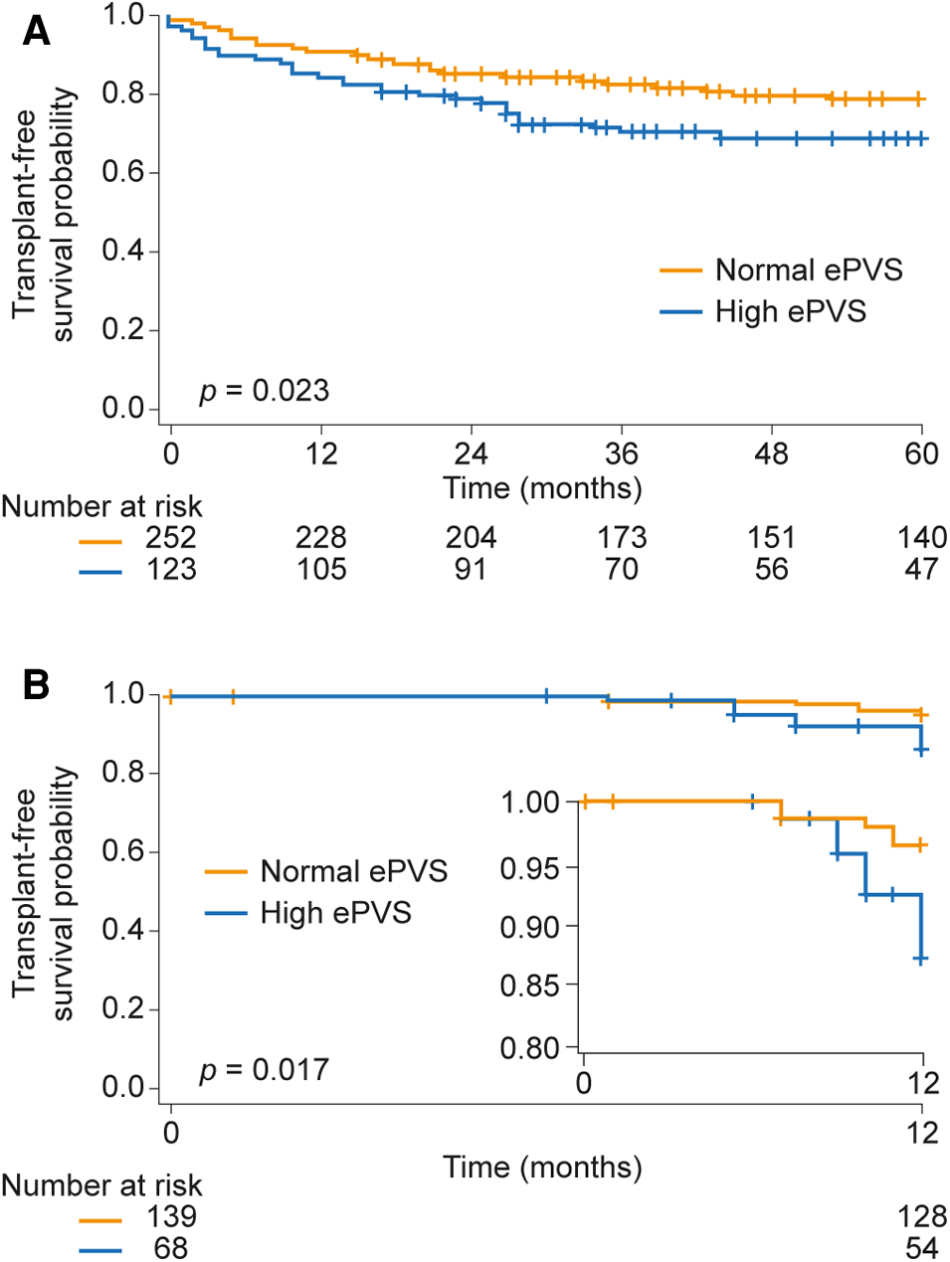
Prognostic relevance of ePVS (**A**) at baseline and (**B**) during follow-up in patients with precapillary pulmonary hypertension. In panel (**B**), the inset shows the same data on an enlarged *y* axis. ePVS, estimated plasma volume status.

Next, Kaplan-Meier analysis was performed to assess the prognostic relevance of ePVS at baseline (Figure 2A). The 1-, 3-, and 5-year transplant-free survival rates of patients with normal ePVS at baseline were 90%, 82%, and 79%, respectively, while patients with high ePVS at baseline showed significantly lower transplant-free survival rates (84%, 70%, and 69%, respectively; log-rank *p* = 0.023). Consistent with this, univariate Cox regression analysis showed a significantly increased hazard ratio (HR) for mortality/transplant (HR per unit increase of baseline ePVS: 1.26 [95% confidence interval (CI): 1.08, 1.47], *p* = 0.004). Regarding non-invasive parameters, BNP concentration, eGFR, TAPSE, inferior vena cava diameter, hematocrit, hemoglobin concentration, and right atrial area were also associated with prognosis in univariate Cox regression as shown in Table 2. Multivariate (stepwise backward) Cox regression showed that ePVS, eGFR, inferior vena cava diameter, and BNP concentration were independently associated with transplant-free survival in patients with precapillary PH (Table 2). Next, we performed a Kaplan-Meier analysis based on thirds of a Cox regression score (regression score $=\sum_{\left(i=1\right)}^{n}coefficient_{i}*parameter_{i}$, for *n* independent predictors of mortality/transplant in backward regression). The 1-, 3-, and 5-year transplant-free survival rates were 98%, 88%, and 88%, respectively, in the low-score group and 91%, 82%, and 78%, respectively, in the intermediate-score group, while the patients in the high-score group had significantly reduced transplant-free survival rates (71%, 52%, and 46% at 1, 3, and 5 years, respectively), as shown in Supplementary Figure S2A.

**Table 2:** Cox regression analysis of the association of baseline parameters with mortality/transplant.

Parameter	Univariate analysis		Multivariate analysis^a^	
	HR (95% CI)	*p* value	HR (95% CI)	*p* value
ePVS	1.256 (1.075, 1.467)	0.004	1.241 (0.960, 1.604)	0.100
BNP	1.001 (1.001, 1.001)	<0.001	1.001 (1.000, 1.001)	0.071
eGFR	0.982 (0.973, 0.991)	<0.001	0.986 (0.970, 1.003)	0.103
TAPSE	0.914 (0.870, 0.960)	<0.001		
Right atrial area	1.032 (1.012, 1.053)	0.002		
Inferior vena cava diameter	1.089 (1.024, 1.158)	0.007	1.069 (0.992, 1.151)	0.081
Hematocrit	0.009 (0.000, 0.400)	0.015		
Hemoglobin	0. 877 (0.795, 0.968)	0.009		

BNP, brain natriuretic peptide; CI, confidence interval; eGFR, estimated glomerular filtration rate; ePVS, estimated plasma volume status; HR, hazard ratio; TAPSE, tricuspid annular plane systolic excursion.

^a^ Stepwise backward model chosen based on Akaike information criteria.

### 3.5. Prognostic relevance of ePVS in prevalent patients with PH

Follow-up data were available for 221 patients. The median follow-up time was 8 [6, 9] months. Patient characteristics at follow-up are presented in Table 3. At follow-up, PAWP, CVP, and BNP concentration were significantly elevated and eGFR was significantly decreased in patients with high ePVS compared with patients with normal ePVS, although the prevalence of edema did not differ significantly between the two groups. TAPSE, inferior vena cava diameter, and right atrial area at follow-up also showed no difference between the two groups. In addition, ePVS showed moderate correlation with CVP (spearman rho = 0.32, *p* = 0.005).

**Table 3:** Follow-up characteristics.

Parameter	All (*n* = 221)	Normal ePVS (*n* = 148)	High ePVS (*n* = 73)	*p* value
Age, years	68 [55, 76] *n* = 221	66 [52, 75] *n* = 148	69 [58, 76] *n *= 73	0.187
Female sex, *n* (%)	130 (59%) *n* = 221	72 (49%) *n* = 148	58 (80%) *n* = 73	<0.001
Diagnosis of IPAH, *n* (%)	108 (49%) *n* = 221	80 (54%) *n* = 148	28 (38%) *n* = 73	0.040
Edema, *n* (%)	59 (27%) *n* = 154	39 (26%) *n* = 108	20 (27%) *n* = 46	0.462
Diuretic treatment, *n* (%)	134 (61%) *n* = 177	94 (64%) *n* = 125	40 (55%) *n* = 52	0.959
Hemoglobin, g/dl	13.5 [12.1, 14.8] *n* = 221	14.5 [13.5, 15.5] *n* = 148	11.5 [10.6, 12.0] *n* = 73	<0.001
Hematocrit	0.41 [0.38, 0.44] *n* = 221	0.43 [0.41, 0.46] *n* = 148	0.35 [0.33, 0.38] *n* = 73	<0.001
Weight, kg	76 [67, 85] *n* = 106	76 [68, 85] *n* = 79	75 [66, 82] *n* = 27	0.442
Height, cm	169 [163, 175] *n* = 201	170 [164, 178] *n* = 134	167 [160, 172] *n* = 67	0.003
mPAP, mmHg	42 [35, 51] *n* = 76	40 [34, 48] *n* = 47	43 [38, 54] *n* = 29	0.089
PAWP, mmHg	10 [6, 13] *n* = 76	8 [6, 12] *n* = 47	11 [9, 15] *n* = 29	0.015
Pulmonary vascular resistance, dyn·s/cm^5^	561 [386, 679] *n* = 76	521 [385, 663] *n* = 47	611 [409, 686] *n* = 29	0.438
CVP, mmHg	7 [4, 10] *n* = 76	6 [4, 9] *n* = 47	9 [7, 12] *n* = 29	0.002
Cardiac index, L/min/m^2^	2.48 [2.13, 2.98] *n* = 76	2.42 [2.12, 2.87] *n* = 47	2.56 [2.13, 3.06] *n* = 29	0.630
Mixed venous oxygen saturation, %	65 [59, 69] *n* = 76	66 [63, 70] *n* = 47	63 [56, 68] *n* = 29	0.008
ePVS, ml/g	4.37 [3.80, 5.16] *n* = 221	3.97 [3.44, 4.37] *n* = 148	5.58 [5.17, 6.42] *n* = 73	<0.001
BNP, pg/ml	114 [44, 259] *n* = 192	98 [32, 245] *n* = 129	147 [74, 329] *n* = 63	0.010
eGFR, ml/min/1.73 m^2^	69 [50, 89] *n* = 106	75 [55, 94] *n* = 79	56 [44, 77] *n* = 27	<0.001
TAPSE, mm	20 ± 4 *n* = 199	20 ± 4 *n* = 133	20 ± 4 *n* = 66	0.183
TAPSE/PASP, mm/mmHg	0.340 [0.247, 0.500] *n* = 181	0.356 [0.263, 0.500] *n* = 120	0.310 [0.232, 0.510] *n* = 51	0.101
Right atrial area, cm^2^	18 [15, 23] *n* = 194	18 [15, 23] *n* = 129	17 [14, 23] *n* = 65	0.458
Inferior vena cava diameter, mm	19 ± 5 *n* = 81	19 ± 6 *n* = 52	20 ± 5 *n* = 29	0.824

Data are presented as median [Q1, Q3] or mean ± standard deviation unless otherwise specified. BNP, brain natriuretic peptide; CVP, central venous pressure; eGFR, estimated glomerular filtration rate; ePVS, estimated plasma volume status; IPAH, idiopathic pulmonary arterial hypertension; mPAP, mean pulmonary arterial pressure; PASP, pulmonary arterial systolic pressure; PAWP, pulmonary arterial wedge pressure; TAPSE, tricuspid annular plane systolic excursion.

High ePVS during follow-up (defined as >4.8 ml/g) was associated with significantly reduced transplant-free survival in a Kaplan-Meier analysis truncated at 12 months (Figure 2B; log-rank *p* = 0.017), and ePVS predicted short-term (1-year) mortality/transplant in univariate Cox regression analysis (HR per unit increase of ePVS during follow-up: 2.12 [95% CI: 1.54, 2.93], *p* < 0.001). BNP concentration, eGFR, TAPSE, hematocrit, and hemoglobin concentration were also associated with transplant-free survival in prevalent patients with PH in univariate Cox regression analysis (Table 4). However, only ePVS, TAPSE, BNP concentration, and eGFR were independently associated with prognosis in multivariate (stepwise backward) Cox regression (Table 4). The resulting Cox regression score showed high concordance (0.912 ± 0.036). The short-term Kaplan-Meier analysis based on thirds of the Cox regression score is shown in Supplementary Figure S2B. The 1-year transplant-free survival rate was 100% and 98% in the low- and intermediate-score groups, respectively, and was significantly compromised (77%) in the high-score group.

**Table 4:** Cox regression analysis of the association of follow-up parameters with mortality/transplant.

Parameter	Univariate analysis		Multivariate analysis^a^	
	HR (95% CI)	*p* value	HR (95% CI)	*p* value
ePVS	2.123 [1.538, 2.930]	<0.001	2.326 [1.492, 3.627]	<0.001
BNP	1.001 [1.000, 1.002]	0.007	0.997 [0.994, 1.000]	0.029
eGFR	0.955 [0.928, 0.993]	0.002	0.974 [0.943, 1.005]	0.104
TAPSE	0.807 [0.707, 0.921]	0.002	0.718 [0.573, 0.900]	0.004
Right atrial area	1.053 [0.996, 1.112]	0.067		
Inferior vena cava diameter	1.081 [0.888, 1.315]	0.439		
Hematocrit	1.087e−08 [6.911e−13, 0.0002]	<0.001		
Hemoglobin	0.568 [0.425, 0.758]	<0.001		

BNP, brain natriuretic peptide; CI, confidence interval; eGFR, estimated glomerular filtration rate; ePVS, estimated plasma volume status; HR, hazard ratio; TAPSE, tricuspid annular plane systolic excursion.

^a^ Stepwise backward model chosen based on Akaike information criteria.

Next, we assessed the prognostic relevance of the change in ePVS from baseline during follow-up in PH. The median change in ePVS was 0.31 [−0.16, 0.87] ml/g. Patients with large decreases in ePVS (below −0.16 ml/g) showed significantly greater decreases in CVP and PAWP than patients with lower decreases in ePVS during follow-up (CVP: −4 ± 5 vs. 0 ± 5 mmHg, *p* = 0.019; PAWP: −2 [−3, −1] mmHg vs. 1 [−1, 4] mmHg, *p* = 0.008). Consistent with these findings, the change in ePVS was associated with prognosis in univariate Cox regression analysis (HR: 1.55 [95% CI: 1.00, 2.38], *p* = 0.048).

### 3.6. Newly described phenotype of high ePVS without edema in patients with incident PH

Of the 317 patients with information available on the presence/absence of edema at baseline, 131 patients (41%) had neither edema nor high ePVS, while 63 patients (20%) had both edema and high ePVS. Interestingly, 39 patients (12%) had high ePVS but no edema. We further characterized this subset of patients.

Patients with high ePVS but no edema had significantly lower mPAP and pulmonary vascular resistance and elevated PAWP, cardiac index, and TAPSE/pulmonary arterial systolic pressure (PASP) ratio compared with patients with normal ePVS and no edema (Table 5). Other pulmonary hemodynamic and echocardiographic parameters were comparable in both groups (Table 5). BNP concentration was not significantly increased. Notably, patients with high ePVS without edema had significantly impaired transplant-free survival, as shown in the Central Illustration. The univariate HR for mortality/transplant in this group compared with patients with normal ePVS without edema was 2.28 (95% CI: 1.18, 4.42). The HR remained significantly increased even after adjustment for age, tricuspid regurgitation, pulmonary vascular resistance, mixed venous oxygen saturation, BNP, and TAPSE/PASP (HR: 2.58 [95% CI: 1.08, 6.17]).

**Table 5:** Characteristics of phenotype subgroups defined by baseline edema and ePVS.

Parameter	No edema, normal ePVS (*n *= 131)	No edema, high ePVS (*n* = 39)	*p* value*	Edema, high ePVS (*n* = 63)	*p* value**
Age, years	65 [51, 75] *n* = 131	69 [57, 76] *n* = 39	0.357	71 [62, 79] *n* = 63	0.246
Female sex, *n* (%)	67 (51%) *n* = 131	21 (54%) *n* = 39	0.909	52 (83%) *n* = 63	0.004
Diagnosis of IPAH, *n* (%)	45 (34%) *n* = 131	16 (41%) *n* = 39	0.567	25 (50%) *n* = 63	1.00
Edema, *n* (%)	0 (0%) *n* = 131	0 (0%) *n* = 39	-	63 (100%) *n* = 63	<0.001
Diuretic treatment, *n* (%)	68 (52%) *n* = 131	24 (62%) *n* = 39	0.381	55 (87%) *n* = 63	0.005
Hemoglobin, g/dl	14.9 [14.1, 16.1] *n* = 131	12.3 [11.8, 12.6] *n* = 39	<0.001	12.0 [11.1, 12.5] *n* = 63	0.030
Hematocrit	0.44 [0.42, 0.48] *n* = 131	0.37 [0.36, 0.38] *n* = 39	<0.001	0.37 [0.34, 0.38] *n* = 63	0.363
Weight, kg	76 [69, 86] *n* = 131	77 [67, 84] *n* = 39	0.530	75 [66, 87] *n* = 62	0.942
Height, cm	170 [164, 178] *n* = 131	172 [164, 176] *n* = 39	0.720	165 [160, 172] *n* = 62	0.073
mPAP, mmHg	42 [33, 50] *n* = 129	37 [29, 44] *n* = 39	0.008	41 [34, 49] *n* = 60	0.054
PAWP, mmHg	8 [6, 12] *n* = 127	10 [7, 14] *n* = 39	0.016	10 [8, 14] *n* = 60	0.484
Pulmonary vascular resistance, dyn·s/cm^5^	636 [430, 878] *n* = 129	389 [255, 613] *n* = 39	<0.001	554 [298, 728] *n* = 60	0.107
CVP, mmHg	6 [3, 9] *n* = 128	6 [5, 9] *n* = 39	0.286	8 [5, 11] *n* = 60	0.063
Cardiac index, L/min/m^2^	2.22 [1.84, 2.51] *n* = 128	2.59 [2.41, 2.97] *n* = 39	<0.001	2.59 [2.02, 2.92] *n* = 60	0.386
Mixed venous oxygen saturation, %	64 [59, 59] *n* = 127	63 [55, 67] *n* = 39	0.123	62 [55, 67] *n* = 60	0.783
ePVS, ml/g	3.77 [3.24, 4.09] *n* = 131	5.08 [4.90, 5.38] *n* = 39	<0.001	5.34 [4.98, 5.88] *n* = 63	0.077
BNP, pg/ml	144 [49, 365] *n* = 118	225 [135, 380} *n* = 34	0.216	252 [13, 536] *n* = 57	0.419
eGFR, ml/min/1.73 m^2^	77 [60, 93] *n* = 131	73 [62, 82] *n* = 39	0.170	56 [38, 75] *n* = 63	0.006
TAPSE, mm	19 ± 5 *n* = 126	20 ± 5 *n* = 37	0.296	19 ± 5 *n* = 61	0.398
TAPSE/PASP, mm/mmHg	0.279 [0.213, 0.380] *n *= 124	0.375 [0.248, 0.473] *n *= 37	0.010	0.288 [0.211, 0.429] *n *= 61	0.070
Right atrial area, cm^2^	19 [15, 24] *n* = 109	19 [16, 25] *n* = 37	0.950	17 [13, 30] *n* = 60	0.970
Inferior vena cava diameter, mm	20 ± 4 *n* = 53	20 ± 6 *n* = 16	0.732	21 ± 6 *n* = 30	0.653

Data are presented as median [Q1, Q3] or mean ± standard deviation unless otherwise specified. BNP, brain natriuretic peptide; CVP, central venous pressure; eGFR, estimated glomerular filtration rate; ePVS, estimated plasma volume status; IPAH, idiopathic pulmonary arterial hypertension; mPAP, mean pulmonary arterial pressure; PASP, pulmonary arterial systolic pressure; PAWP, pulmonary arterial wedge pressure; TAPSE, tricuspid annular plane systolic excursion.

^*^ No edema, high ePVS vs. no edema, normal ePVS.

^**^ Edema, high ePVS vs. no edema, high ePVS.

### 3.7. Reason for development of edema in patients with high ePVS and chronic cardiorenal syndrome

Of 54 patients with high ePVS and chronic cardiorenal syndrome, 45 patients had edema (83%). To investigate the underlying mechanism leading to edema in this subgroup, we performed backward binary logistic regression. Interestingly, only TAPSE/PASP (*B* = −6.34, *p* = 0.066) and sex (*B* = −1.51, *p* = 0.086) were independently associated with edema while age, eGFR, inferior vena cava diameter, right atrial area, pulmonary vascular resistance, cardiac index, mixed venous oxygen saturation, and BNP were not.

## 4. Discussion

Our study assesses the relevance of ePVS in a homogeneous study population of patients with precapillary PH (IPAH and CTEPH). High ePVS was associated with congestion and cardiorenal syndrome and predicted mortality/transplant both at baseline and during follow-up. Furthermore, a decrease in ePVS was associated with a decrease in volume overload (i.e., decongestion) and with improved prognosis. Notably, the prognostic utility of ePVS was independent of other non-invasive biomarkers such as eGFR, BNP concentration, or echocardiographic parameters such as TAPSE and right atrial area. A newly defined phenotype of high ePVS without edema was characterized and its prognostic relevance was demonstrated. Thus, in addition to the approaches currently used, ePVS enables physicians to assess the volume (over)load and prognosis of patients with precapillary PH.

Although the reserve of right ventricular-pulmonary arterial coupling is high in patients with PH, both chronic and acute volume overload result in clinical deterioration accompanied by a worsened prognosis (19, 20). Non-invasive assessment of volume overload in patients with heart failure is therefore crucial, but is complicated by the imprecision of clinical signs such as edema and the lack of accurate biomarkers. Our study shows that ePVS is a feasible tool for estimating central venous congestion in PH, as high ePVS was associated with increased CVP. Notably, right ventricular systolic function (reflected by TAPSE) was not consistently impaired in patients with PH and plasma volume overload. In addition, there was no right atrial dilatation. Therefore, one can speculate that ePVS is an early biomarker of volume overload, before contractility worsens and remodeling occurs in the right side of the heart. In contrast to ePVS, BNP concentrations (currently widely used to assess congestion in patients with PH) are strongly influenced by right ventricular function (21). Furthermore, multivariate (stepwise backward) Cox regression showed that ePVS is an independent predictor of mortality/transplant in incident patients with PH and can therefore be used in addition to the existing (non-invasive) methods of risk assessment in PH (1, 22).

At follow-up examinations, high ePVS was associated with increased central venous congestion (reflected by invasively measured CVP). Similarly, ePVS predicted mortality/transplant in patients with precapillary PH. Once again, ePVS was not meaningfully dependent on right ventricular systolic function and right atrial enlargement. In addition, ePVS remained as an independent predictor of mortality/transplant in multivariate (stepwise backward) Cox regression and can therefore be used as an additional biomarker to assess congestion during follow-up. Notably, concordance of a new risk score including ePVS was high, exceeding 0.900.

Interestingly, decongestion (reflected by an intra-patient decrease in ePVS) was associated with decreases in CVP and PAWP. In addition, the intra-patient decrease in ePVS was a predictor of transplant-free survival. From this, it can be concluded that the intra-individual course of ePVS can be used to monitor the effect and sufficiency of treatment in patients with IPAH or CTEPH.

We have for the first time described a phenotype of high ePVS without edema, which had a high prevalence in our study cohort. Patients with this phenotype showed less severe elevation of mPAP and pulmonary vascular resistance and no significant difference in right ventricular function and BNP concentration compared with patients with normal ePVS without edema. Nevertheless, transplant-free survival was poor and comparable to that in patients with edema and high ePVS, underscoring the clinical relevance of a thorough assessment of plasma volume in patients with precapillary PH. Special attention needs to be paid to this newly described phenotype because it is easily underdiagnosed due to normal echocardiographic right heart morphology and function. The associated risk of mortality is overlooked in risk assessments based on the usual clinical parameters. We thus expect clinical (risk) assessment of patients with precapillary PH to be improved by inclusion of ePVS. However, due to the lack of prospective studies, it remains unclear how patients with this phenotype should be managed and whether escalation of diuretic therapy can increase the probability of transplant-free survival. In patients with high plasma volume and impaired renal function in our study cohort, impaired RV-PA coupling (besides sex) was the main reason for the development of edema. Other parameters, including eGFR, inferior vena cava diameter, right atrial area, right ventricular function, and pulmonary hemodynamics were not associated with edema.

**Figure 3:**
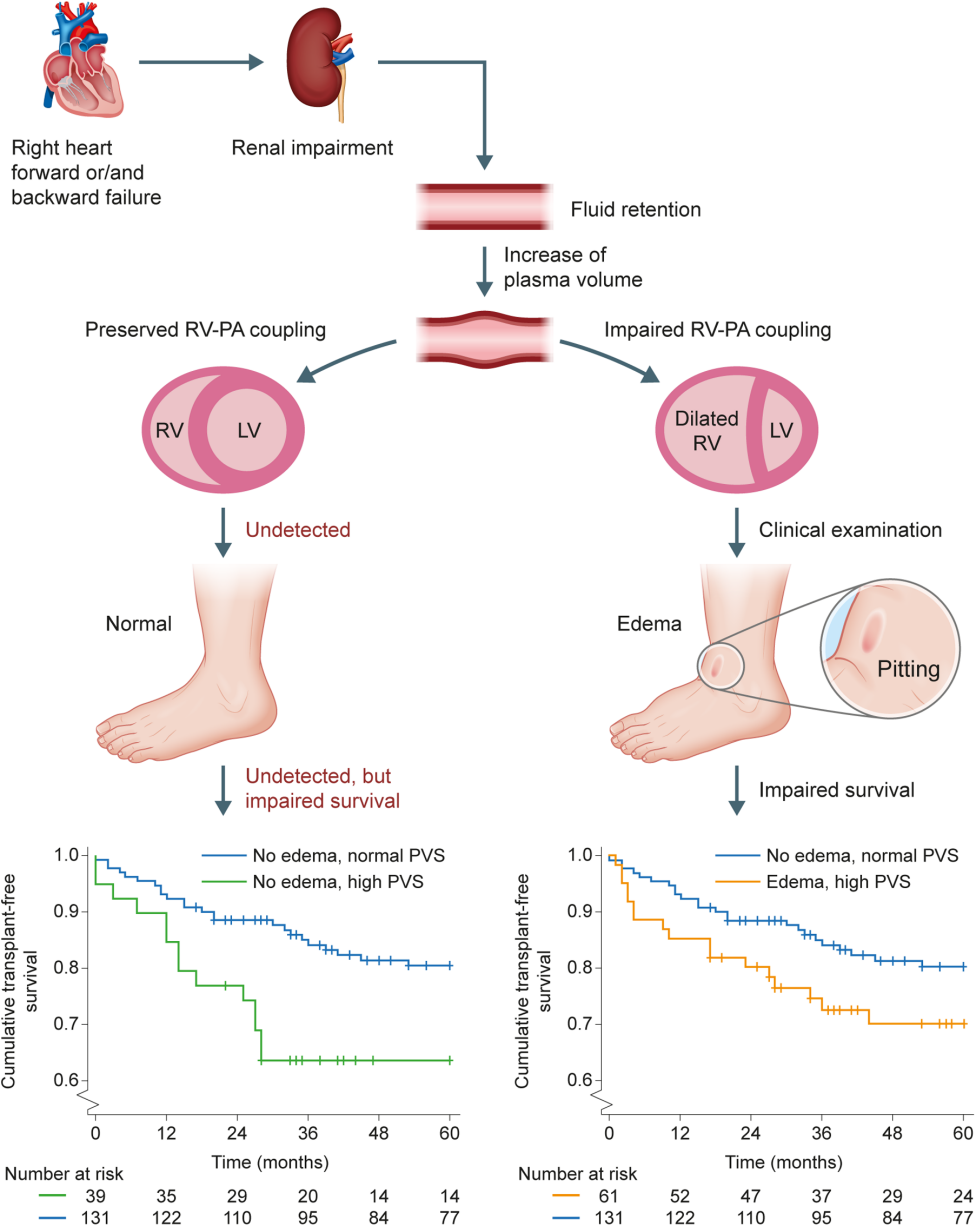
Prognostic relevance of high ePVS in the absence of edema. Forward and backward failure in the right side of the heart both lead to impaired renal function and thus to fluid retention. Patients with impaired right ventricular function show edema due to high plasma volume and have impaired transplant-free survival (lower Kaplan-Meier plot). Patients with preserved right ventricular function do not show edema but still have a high plasma volume (undetected in routine clinical examination) and significantly impaired transplant-free survival (upper Kaplan-Meier plot; *p* = 0.012). In both Kaplan-Meier plots, the comparator is the group of patients with normal ePVS without edema. ePVS, estimated plasma volume status; LV, left ventricle; PA, pulmonary artery; RV, right ventricle.

From a pathophysiological point of view, it is known that an increased right ventricular afterload leads to both venous congestion and reduced forward stroke volume (4, 19, 23). Both lead to impaired renal function and thus to fluid retention (Figure 3) (23). Patients with impaired right ventricular function show edema due to high plasma volume. These patients can be identified through clinical examination and treated appropriately. Conversely, patients with preserved right ventricular function do not show edema but still have an increased plasma volume. These patients cannot be identified through routine clinical examination because their clinical and echocardiographic findings appear normal, although they have a significantly reduced survival time.

It should be noted that hemoglobin concentration and hematocrit—which are included in the calculation of ePVS (8, 24)—depend not only on plasma volume, but also on constellations driving erythropoiesis such as chronic hypoxemia or PH-targeted therapy during follow-up (e.g., endothelin receptor antagonists causing anemia), in particular in patients with PH and (right-sided) heart failure (25). Under these circumstances, ePVS decreases since the hemoglobin concentration is in the denominator of the formula for estimating plasma volume (8). By including hematocrit in the multivariate regression analysis, our study gives evidence for the superiority of ePVS. In this context, it is also important to consider that the ePVS formula has shown a good correlation with the measured plasma volume in patients with heart failure and preserved ejection fraction (7). Nevertheless, ePVS must always be evaluated in the overall clinical context and should not be considered as a stand-alone parameter.

Our study is limited due to its retrospective study design. Missing data and subsequent exclusion of patients may have resulted in significant bias. Furthermore, the usefulness of ePVS for guiding treatment strategy/diuretic escalation remains unclear. Chronic cardiorenal syndrome was solely defined by eGFR without exclusion of other reasons which may have led to increased creatinine concentrations, owing to the retrospective study design. The definitive mechanism leading to the association of ePVS with congestion and cardiorenal syndrome cannot be directly assessed due to the retrospective nature of the study. Therefore, a prospective assessment is warranted.

In conclusion, we believe that the estimation of plasma volume status may be a feasible additional method to assess and monitor volume overload and congestion in patients with precapillary PH. It is a novel finding that elevated ePVS values are linked with poor survival, even in the absence of right heart decompensation and overt edema formation. Owing to its added prognostic value in combination with existing non-invasive biomarkers, ePVS is a promising clinical assessment tool.

## Data Availability

The raw data supporting the conclusions of this article will be made available by the authors, without undue reservation.
