# Supplementary material for: Estimated plasma volume status: association with congestion, cardiorenal syndrome and prognosis in precapillary pulmonary hypertension
Source: Front Cardiovasc Med. 2023 May 10;10:1161041. doi: 10.3389/fcvm.2023.1161041 (PMC10206211; doi:10.3389/fcvm.2023.1161041)
Supplement: Supplementary file 1 [file Datasheet1.docx]

# Supplementary Figures


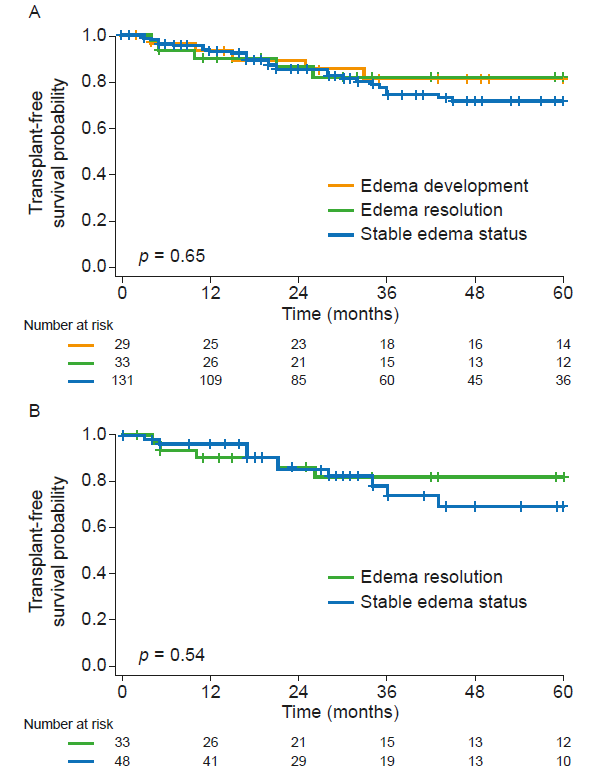


**FIGURE S1** Development or resolution of edema is not prognostically relevant in **(A)** the study population and **(B)** the subgroup of patients who presented with edema at baseline.


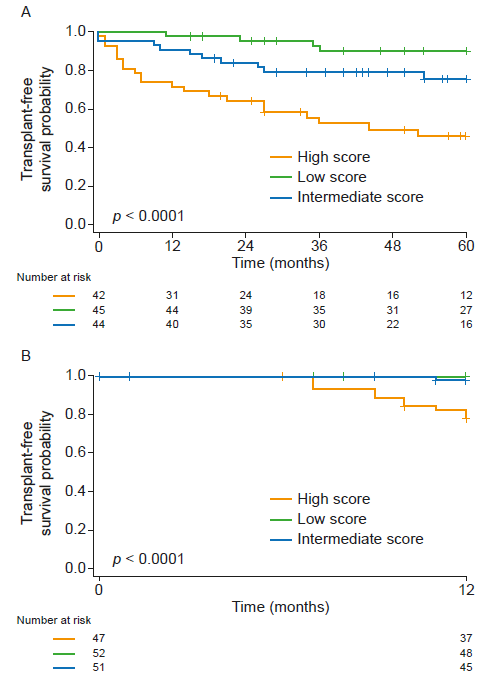


**FIGURE S2** Kaplan-Meier analysis of patients stratified based on a Cox regression score calculated using **(A)** baseline data and **(B)** follow-up data.
